# Complex Regional Pain Syndrome Caused by an Axillary Lipoma

**DOI:** 10.7759/cureus.12280

**Published:** 2020-12-25

**Authors:** Raj H Patel, Rishi Sheth, Nir Hus

**Affiliations:** 1 Surgery, Edward Via College of Osteopathic Medicine, Monroe, USA; 2 Surgery, Florida Atlantic University Charles E. Schmidt College of Medicine, Boca Raton, USA; 3 Surgery, Florida Atlantic University, Boca Raton, USA; 4 Surgery, Delray Medical Center, Delray Beach, USA

**Keywords:** complex regional pain syndrome, surgery, brachial plexus, lipoma, reflex sympathetic dystrophy

## Abstract

Complex regional pain syndrome (CRPS), formerly known as reflex sympathetic dystrophy syndrome, is a rare chronic neuro-inflammatory pain condition, which can follow a soft-tissue, bone (type I), or nerve injury (type II) that can be severe and often lasts longer than the original tissue damage. Lipomas impinging on the brachial plexus are rare. To date, there have been no documented cases of CRPS caused by a benign tumor. Here, we report a rare case of CRPS caused by surgical removal of a left axillary lipoma impinging on the brachial plexus. The patient presented with neuropathic pain and hyperalgesia of the left arm, in a non-dermatomal pattern, and pain out of proportion to touch and painful stimulus. Persistent CRPS continued to occur post-operatively for one year without significant change in her pain characteristics. CRPS following elective or emergent surgery to the extremities can pose significant complications to recovery and post-operative care. This condition can be induced through surgery or trauma, which can complicate recovery, impair motor functionality, and cause debilitating pain. Treatment modalities and pathogenesis for CRPS remain obscure and limited, which leads to wide misdiagnosis. Our case highlights the importance of considering CRPS when evaluating differential diagnoses for pre- and post-operative conditions affecting the upper and lower extremities.

## Introduction

Complex regional pain syndrome (CRPS) is a rare form of neuropathy characterized by severe pain, edema, and vasomotor disturbances, which can affect one or more extremities of the body [1]. Although its pathogenesis and etiology remain unclear, CRPS has been thought to originate from an abnormal dysregulation of both the central and peripheral nervous systems. CRPS generally occurs secondary to clear history of trauma or injury. The most common injury associated with CRPS is a fracture, which occurs in more than 40% of CRPS cases [1]. Other forms of inducing trauma or injury may include sprains, contusions, or surgery. CRPS is divided into two subtypes: CRPS type I and type II. Type I, formerly known as reflex sympathetic dystrophy syndrome, occurs in the absence of nerve injury [2]. Type II, formerly known as causalgia, occurs in the presence of nerve injury.

Clinical diagnosis can be challenging as both types of CRPS follow a regional pain distribution rather than a dermatomal or peripheral nerve pattern [3]. CRPS is further subdivided into other subtypes of "warm" and "cold", and sympathetically maintained versus sympathetically independent, which may affect prognosis and treatment options [4]. The condition manifests primarily through the autonomic, motor, or trophic bodily changes. Post-operative diagnosis of CRPS can complicate recovery and impair the functional and psychological well-being of patients [5]. Here, we present a case of a 91-year-old woman with symptoms of CRPS who underwent surgery for removal of a left axillary lipoma and decompression of the left brachial plexus. She presented with neuropathic pain and hyperalgesia of the left arm, in a non-dermatomal pattern, and pain out of proportion to touch and painful stimulus. This unique clinical presentation persisted for one year following the complete removal of the lipoma.

## Case presentation

Presentation

A 91-year-old patient was referred to our neurosurgical clinic with a history of severe erratic left arm pain and swelling for three months. The patient reported intermittent weakness of the left arm and pain radiating from the proximal shoulder down to her left fifth digit.

**Figure 1:**
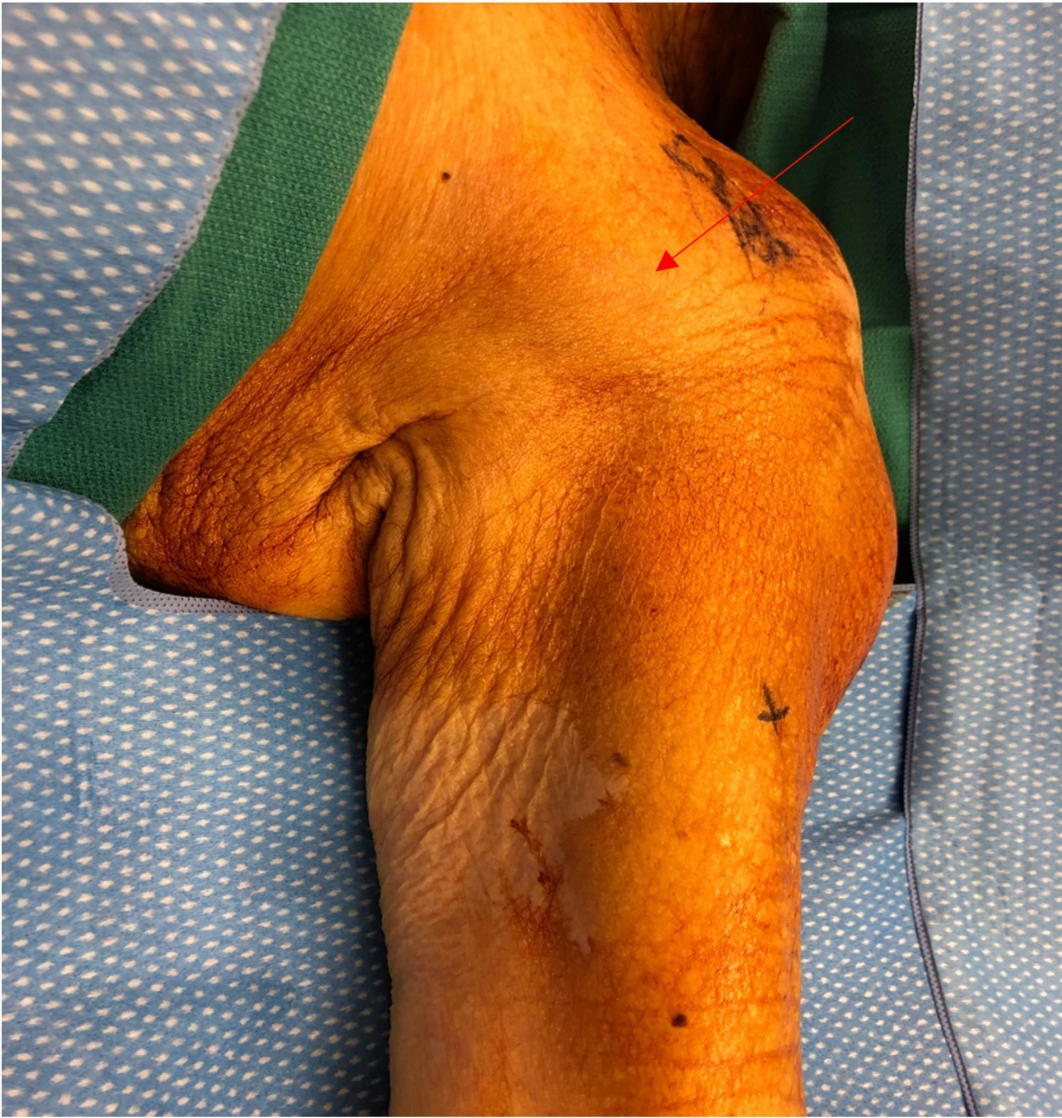
Left brachial plexus region Red arrow indicates the location of the axillary lipoma prior to operation.

Physical examination revealed acute distress due to pain with left arm guarding. The patient appeared uncomfortable and frequently changed her position for relief and complained of hyperalgesia in a non-dermatomal pattern upon palpation of the forearm or hand. The pain was out of proportion to the stimulus of pinprick or touch. A palpable mass deep in the left axilla with severe tenderness to deep palpation and pressure was found (Figure 1). Generalized hyper-paresthesia and decreased motor strength throughout the left arm were also noted. Testing motor strength was limited as a result of pain. The handgrip was 3/5 with severe pain in the left arm. Past medical history was unremarkable except for a prior surgical history of a hip replacement.

Magnetic resonance imaging (MRI) with and without contrast of the left brachial plexus revealed a large mass in the left axilla measuring 4.2 x 3.0 cm transverse and 7.8 cm proximal to distal superficial to the subscapularis but deep to the coracobrachialis and short head of the biceps. An MRI of the left shoulder showed the large simple-appearing lipoma displacing the components of the brachial plexus within the axilla. The patient agreed to proceed with surgery to remove the axillary lipoma.

Operation

**Figure 2:**
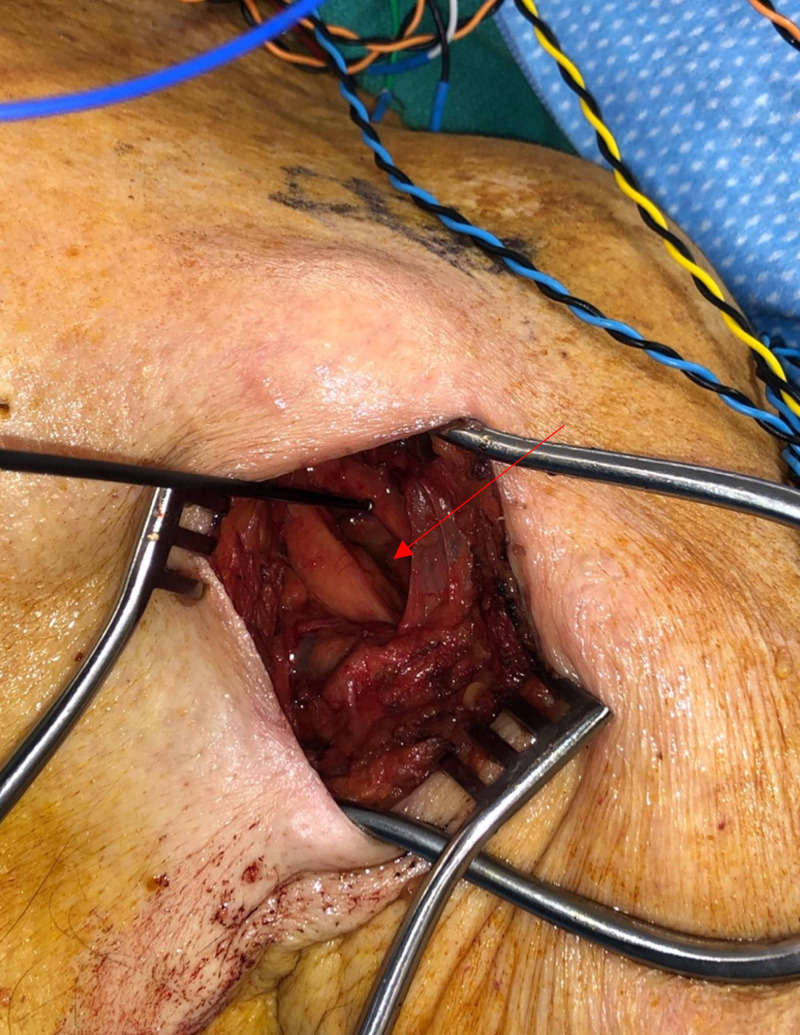
Operative Image Image showing intraoperative neurophysiological monitoring with stimulation probe during surgery. Red arrow shows the location of the axillary lipoma after removal.

The left axillary lipoma was removed, and axillary contents were explored along with decompression of the brachial plexus. The patient was placed in a supine position with the left arm abducted. After prepping and draping the patient, a subclavicular and lateral axillary linear incision was made. Dissection was carried until a compressible mass was encountered around the brachial plexus structure (Figure 2). Care was taken to preserve the nerve roots and vessels. Frequent stimulation of the nerve roots was conducted by using a probe during the procedure. The brachial plexus nerve roots surrounded the lipoma and were stretched by the lipoma. The brachial plexus structures were found to be attached to the capsule of the lipoma. The nerve roots were easily peeled off the lipoma capsule, and there was no infiltration of the nerve roots or surrounding tissue. The lipoma was removed and sent for pathologic evaluation. The nerve roots and brachial plexus were stimulated at the end of the procedure, confirming the integrity of the nerve roots. The patient tolerated the procedure well without any complications.

Postoperative course and histopathology

**Figure 3:**
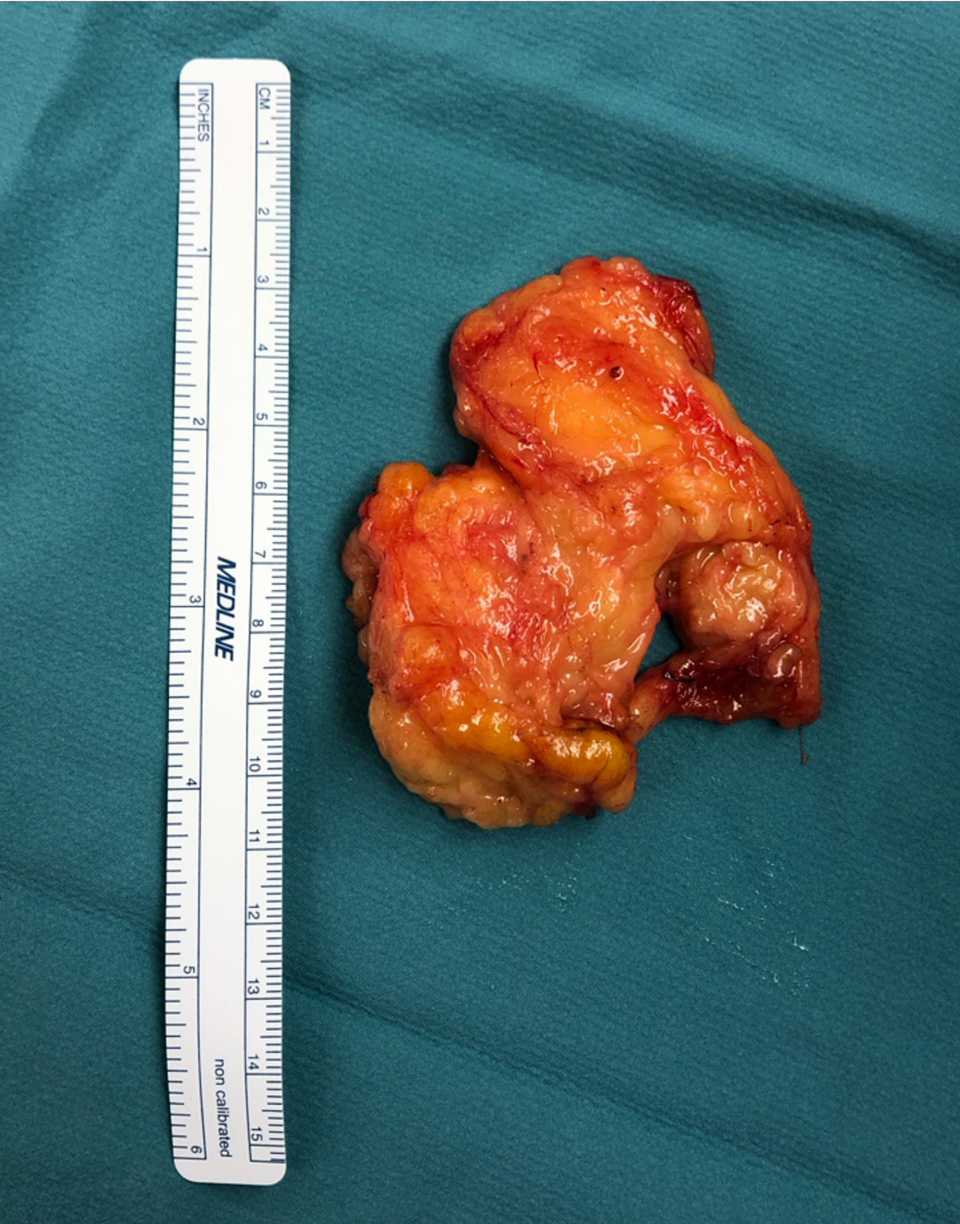
Excised left axillary lipoma Axillary lipoma measuring 7.3 x 5.9 x 1.5cm excised in one piece.

Two weeks postoperatively, the patient’s symptoms in the left arm had improved but were not completely resolved. The resected mass measured 7.3 x 5.9 x 1.5 cm and was confirmed by pathology analysis to be a lipoma with no evidence of any malignancies (Figure 3). Four weeks postoperatively, the patient presented with neuropathy and pain in the left arm, which was out of proportion to any stimulus. She complained of sporadic and patchy pain in the left fifth digit, medial side of the forearm at the elbow, and in the medial upper arm. The pain did not follow any dermatomal distribution and was exacerbated upon lifting her arm. A referral to a pain management clinic for evaluation and treatment of the CRPS was requested. Computed tomography of the chest and complete X-ray of the left shoulder were performed and were unremarkable. The patient was advised to consider CRPS treatment and to follow-up with her neurologist for treatment and management of neuropathic pain. A pain management physician confirmed the diagnosis of CRPS symptoms and noted aggravated symptoms with exposure to cold temperature. Temporary relief was obtained with prescribed narcotics. 

Based on a one-year postoperative follow-up conversation with the patient, she continued to have unresolved neuropathy and hyperalgesia in the left arm. Her pain stems from the proximal left shoulder to the posterolateral elbow. She also reports difficulty in lifting the left arm due to the onset of excruciating pain but has no muscle spasms, tremors, numbness, or weakness of the left arm. The pain was moderately controlled with gabapentin and oxycodone, allowing the performance of activities of daily living.

## Discussion

Lipomas of the brachial plexus are rare. Although most lipomas are asymptomatic, some can be a significant source of pain and weakness due to their slow but progressive growth near peripheral nerves. Previous reports show that lipomas can cause compression neuropathy due to mass effect when found near peripheral nerves [6]. Surgical excision of brachial plexus lipomas should be considered if they are symptomatic and progressively increase in size. In these instances, careful resection of lipomas in the brachial plexus region is critical to prevent nerve injury. Our patient had an unusual diagnosis and symptoms of complex regional pain syndrome (CRPS) prior to surgery, caused by a large axillary lipoma, which was thought to be causing a cascade of symptoms by impinging on the peripheral nerves through a mass effect [7]. Her diagnosis of CRPS was strongly supported by her pre- and postoperative clinical presentation. Two weeks postoperatively, pain symptoms improved but plateaued, and the original clinical presentation of CRPS continued after one-year following surgery. The complex and unclear pathophysiology behind CRPS can pose a significant challenge to accurate clinical diagnosis and can hinder perioperative management. To our knowledge, this is the first known clinical presentation of a benign lipoma causing symptoms of CRPS. 

Complex regional pain syndrome is a debilitating condition accompanied by motor and autonomic dysfunction, occurring secondary to surgical trauma or injury to the nerve or soft tissue. Although the pathophysiology behind CRPS is multifactorial and remains unclear, it is thought to originate from an over-reaction of highly sensitized pain extending from peripheral nociception. This, in turn, disrupts the body's autonomic response, which leads to abnormal activation of the sympathetic nervous system [8]. CRPS has been found to occur after limb surgery, fractures, or injuries to one or more extremities [7]. The classic clinical course of complex regional pain syndrome (CRPS) includes various symptoms such as hyperalgesia, neuropathy, vasomotor dysfunction, and psychological disorders, which are pathologically defined by abnormal activation of the nervous system [9]. Generally, pain levels are out of proportion to the injury or trauma and present in a non-anatomic distribution, as observed in our patient. Long-standing pressure directly on the nerves, due to lipoma growing slowly within a confined space, may initiate a cascade of events leading up to neuropathic pain.

Although most cases tend to resolve within a year following the inciting trauma, some cases progress to a more severe, chronic form of the condition [10]. Development of post-surgical CRPS is not uncommon; however, little is known regarding predisposing risk-factors to surgical candidates for CRPS [11]. Surgical procedures that may pose a greater risk for CRPS and optimal perioperative treatment options for patients are also unknown. Surgery to extremities that have been previously affected with CRPS is generally avoided due to the risk of recurrence or worsening of symptoms; however, a stellate ganglion block or perioperative sympathectomy can be beneficial for CRPS patients requiring surgery [12]. Surgical decision making can become particularly challenging in complex cases such as ours in which the patient’s original symptoms of CRPS went unidentified due to the presentation of the axillary lipoma.

Differential diagnoses for CRPS may include sensorimotor neuropathy, erythromelalgia, vascular insufficiency, lymphedema, deep vein thrombosis, and Reynaud’s phenomenon [1]. The fast progression of symptoms and clinical similarities with differential diagnoses makes CRPS a challenging diagnosis. Treatment options primarily include a range of therapeutic techniques depending on the severity and type of symptoms, which can yield ephemeral benefits. Some of these options include corticosteroids, anticonvulsants such as gabapentin, low-dose tricyclic antidepressants, transdermal lidocaine, opioids, chemical sympathetic ganglion blocks, physical therapy, or neuromodulation [1]. Due to the close association of CRPS with psychological abnormalities, cognitive behavioral therapy can also be considered as a treatment option for patients [13]. CRPS can greatly impact the quality of life due to the extended duration of pain in the affected area and the ability to continue routine activities. Spontaneous improvement of CRPS patients has been previously reported; however, given the unbearable and incapacitating nature of symptoms, it is advisable to begin therapy and management of symptoms as soon as possible to avoid muscular atrophy and contracture [14]. Most CRPS cases tend to resolve within the first year, with a very small fraction of cases progressing to the chronic form [15]. Timely intervention by a chronic pain management specialist can also play a vital role in having a favorable prognosis.

## Conclusions

Lipomas rarely affect peripheral nerves and have not been previously reported to cause or be associated with CRPS. To our knowledge, this is the first known clinical presentation of a benign lipoma causing symptoms of CRPS. It is vital for surgeons to consider CRPS when evaluating differential diagnoses for pre- and postoperative conditions affecting the upper and lower extremities. Cautious and early diagnosis of complex regional pain syndrome is critical in assuring patients' favorable outcome and avoiding surgery. The presence of axillary lipomas can hinder perioperative management for CRPS patients and can pose a challenge to early diagnosis. The lack of randomized controlled clinical trials and guidelines for optimal management of CRPS in the surgical setting poses significant challenges in choosing an optimal course of therapy for these patients. Although prognosis is difficult to predict, an early diagnosis and intervention can increase the likelihood of a favorable outcome. Treatment options for CRPS should be considered based on the severity of patient symptoms and can include a combination of physical therapies, analgesics, or sympathetic blockades for severe cases.
